# Supplementary figures and images for: Compositional clustering in task structure learning
Source: PLoS Comput Biol. 2018 Apr 19;14(4):e1006116. doi: 10.1371/journal.pcbi.1006116 (PMC5929577; doi:10.1371/journal.pcbi.1006116)

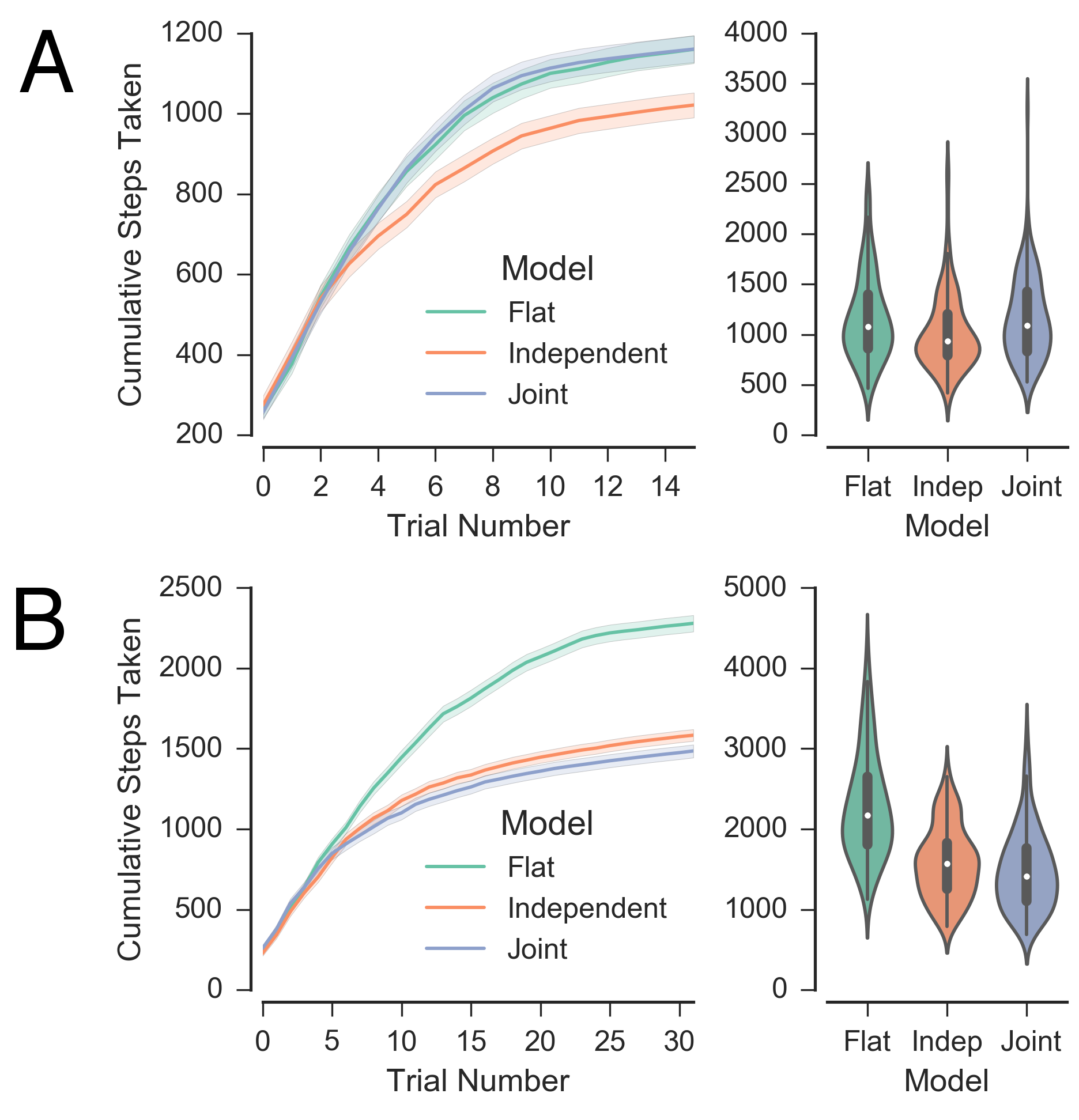

Supplement: S1 Fig — A. Agents’ performance learning full transition function in Simulation 1. A, Left: Cumulative number of steps taken by each model as a function of trials. Fewer steps represents better performance. A, Right: Distribution of total number of steps required to complete the task for each agent. B. Agents’ performance function in Simulation 2. (TIF) [file pcbi.1006116.s001.tif]

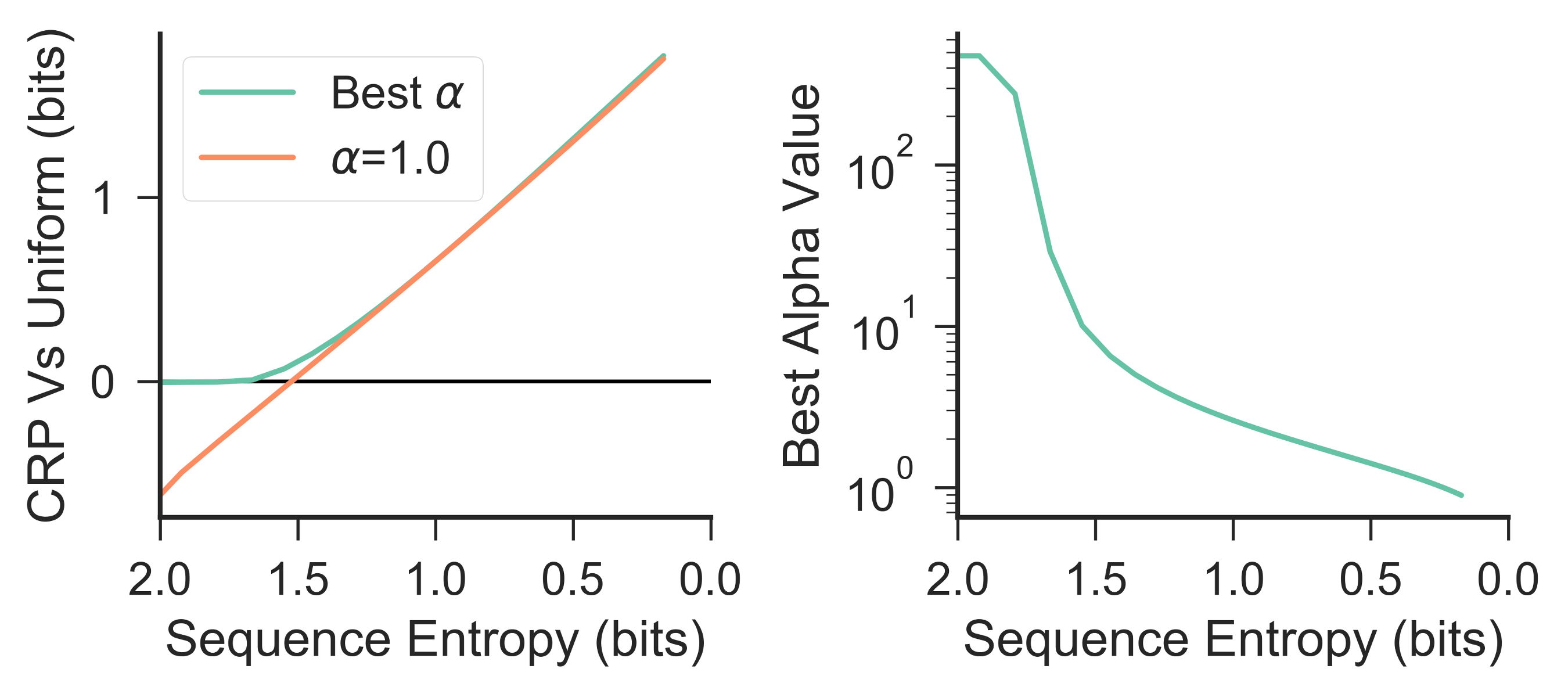

Supplement: S2 Fig — Left: Relative information gain of a naïve guess over the CRP as function of sequence entropy for a CRP with an optimized alpha parameter (green) or fixed at α = 1.0. Right: Optimized alpha value (log scale) as a function of sequence entropy. (TIF) [file pcbi.1006116.s002.tif]
