# Supplementary material for: Compositional clustering in task structure learning
Source: PLoS Comput Biol. 2018 Apr 19;14(4):e1006116. doi: 10.1371/journal.pcbi.1006116 (PMC5929577; doi:10.1371/journal.pcbi.1006116)
Supplement: S1 Text — (PDF) [file pcbi.1006116.s003.pdf]

S1: Grid world simulations with unknown transition functions

Here, replicate simulations 1 and 2 presented in the main article while relaxing the the assumptions the agent knows the spatial relationship between states. Instead, we consider the case in which an agent needs to learn the full transition functions over states, actions and successor states. The simulations presented here are the same as simulations 1 and 2 in the main article but we redefine the agents such that they no longer have access to the the transition function in terms of cardinal movements $T_c(x, A, x')$.

We define the transition functions learned by the agents in terms of primitive actions $a \in \mathcal{A}$ as $f_c(x, a, x')$ in context c by marginalizing over cardinal actions such that

$$\begin{aligned} f_c(x, a, x') &= \sum_{A \in \mathcal{A}_{card}} \phi_c(a, A) T_c(x, A, x') \\ &= \Pr(x'|x, a, c) \end{aligned} \tag{S1}$$

Correspondingly, the optimal policy can also be re-expressed in in terms of transition function f_c as:

$$\pi_c^*(x) = \arg\max_a \left[\sum_{x' \in X} f_c(x, a, x') \left[R_c(x') + \gamma V_c(x') \right] \right] \tag{S2}$$

Likewise, the optimal value function is thus:

$$V_c(x) = \max_a \left[\sum_{x' \in X} f_c(x, a, x') \left[R_c(x') + \gamma V_c(x') \right] \right] \; \forall \; x \in X \tag{S3}$$

For the purpose of generalization, we assume the agents generalize the full transition functions, as opposed to generalizing the mapping functions presented in the main manuscript. As $T_c(x, A, x')$ was previously assumed to be known, generalizing mappings can be seen as a specific case of generalizing full transition functions as a consequence of equation S1.

For the purpose of clustering contexts, the mapping function was used as a component of the likelihood function. Specifically, the likelihood function for the context-clustering assignments in joint clustering is the product of the mapping and reward functions $L(\mathcal{D}|k) = \phi_k(a, A) R_k(x, A)$. Independent clustering assigns contexts into clusters separately for mappings and rewards, using $L(\mathcal{D}|k_\phi) = \phi_k(a, A)$ as the mapping cluster likelihood. As a consequence of equation S1, we can create the more general case of these two likelihood functions, respectively, with the following:

$$L(\mathcal{D}|k) = f_k(x, a, x') R_k(x') \tag{S4}$$

$$L(\mathcal{D}|k_\phi) = f_k(x, a, x') \tag{S5}$$

No further changes in the generative framework are needed to accommodate clustering full transition functions.

In the following simulations, agents estimated $\hat{f}_k(x, a, x') = \hat{\Pr}(x'|x, a, c \in k)$ with maximum likelihood estimation, assuming independence between $f_k(x, a, x')$ for all values of x and a. Action selection was performed by a combination of Thompson sampling [1] and ϵ-greedy exploration. For joint clustering, a single context-clustering hypothesis was sampled on each time step proportionally to its posterior probability. The estimates $\hat{f}_k$ and $\hat{R}_k$ from sampled hypothesis were used to compute a state-action value function, defined

$$\hat{Q}_k(x, a) = \hat{R}_k(x') + \gamma \sum_{x' \in X} f_k(x, a, x') \hat{V}_k(x') \tag{S6}$$

where the value function $V_k(x')$ is generated from equation S3] via dynamic programming [2]. The sampled state-action value function is generated in the same manner for independent clustering, except that $\hat{f}_k$ and $\hat{R}_k$ are sampled from the separate sets of clusters. Likewise, the flat model assumes the singular hypothesis that each context belongs to its own cluster and thus always samples the same hypothesis.

The state-action value function is used to generate a policy via an epsilon-greedy exploration rule where the action with the highest value $\hat{Q}(x = x_t, a)$ was chosen with probability $1 - \epsilon$ and a random action was chosen with probability ϵ (ties were broken with equal probability).

Simulation 1

We first simulated the three agents on the same 150 random tasks presented in Simulation 1 of the manuscript. In this set of simulations, each of two reward functions and each of two transition functions were repeated across four contexts such that each context had a unique combination of reward and transition functions. The models were simulated using the parameter values $\gamma = 0.75$, $\epsilon = 0.5$ and $\alpha = 1.0$. As in the main manuscript, the independent clustering model learned the task more quickly than either of the other two models ($p < 0.003$ vs. joint; $p < 0.004$ vs. flat), completing all trials in an average of 1021.0 steps (s=377.2) in comparison to 1160.3 (s=408.3) and 1159.7 (s=424.9) steps for the the joint clustering and flat agents, respectively (S1 Fig, A).

S1 Fig. *A*: Agents' performance learning full transition function in Simulation 1. *A, Left*: Cumulative number of steps taken by each model as a function of trials. Fewer steps represents better performance. *A, Right*: Distribution of total number of steps required to complete the task for each agent. *B*: Agents' performance function in Simulation 2.

Simulation 2

We then simulated the three agents on the same 150 random tasks presented in Simulation 2 of the manuscript. In this set of simulations, each of four reward functions and each of four transition functions were repeated across eight contexts such that each paring of reward and transition functions was repeated across two contexts. As in the main manuscript, the joint clustering model learned the task more quickly than either of the other two models ($p < 0.08$ vs. independent; $p < 10^{-26}$ vs. flat), completing all trials in an average of 1485.8 steps (s=496.4) in comparison to 1583.6 (s=449.1) and 2820.0 (s=653.8) steps for the the independent clustering and flat agents, respectively (S1 Fig, B).

References

1. Thompson WR. On the likelihood that one unknown probability exceeds another in view of the evidence of two samples. Biometrika. 1933 Dec 1;25(3/4):285-94.

2. Sutton RS, Barto AG. Reinforcement learning: An introduction. Cambridge: MIT press; 1998 Mar 1.
