# Supplementary material for: Compositional clustering in task structure learning
Source: PLoS Comput Biol. 2018 Apr 19;14(4):e1006116. doi: 10.1371/journal.pcbi.1006116 (PMC5929577; doi:10.1371/journal.pcbi.1006116)
Supplement: S2 Text — (PDF) [file pcbi.1006116.s004.pdf]

S2: CRP performance as a function of task structure

One question we can ask is how well the CRP prior generalizes as a function of the underlying structure of the task domain. Intuitively, we might expect that a generalization agent should show a generalization benefit in highly structured task domains and show a less of a generalization benefit in unstructured domains. We can thus define a set of restricted task domains that vary in their structure. Specifically, we can vary the predictability of the generative process p and evaluate the agent as a function of this predictability.

Let $\mathcal{K} = \{A, B, C, D\}$ be the set of possible clusters in a task domain with probabilities $\mathcal{P} = \{p_A, p_B, p_C, p_D\}$. Let $X = ABCD$ represent a sequence of contexts and cluster identities experienced by an agent, such that cluster A is experienced in context c_1, B is experienced in context c_2, etc. We assume that the cluster identity is observable from the statistics of the associated MDP and that the agent knows the members of the set $\mathcal{K}$.

We want to evaluate the ability of the CRP prior to predict each cluster in the sequence, conditional on its own history. We do so by calculating the expected loss experienced by the CRP over the sequence X. We define our loss function over sequence X as

$$L(p, f(X)) = -\frac{1}{||X||} \sum_X \log_2 q_k^{(t)} \tag{1}$$

where $q_k^{(t)}$ is the CRP's probability estimate for the value $k = X_t$ given $X_{1:t-1}$. As noted above, this loss function is equivalent to the cross entropy $H(q, p)$ between the CRP and the generative process. That is, $H(q, p)$ is the average degree of unpredictability (in bits of information content) of experiencing each MDP given the estimate q. We can assess $H(q, p)$ for the CRP by updating the predictive distribution in each context and probing its estimate for the subsequent context. As the CRP is exchangeable [1], $H(q, p)$ is invariant to the order of the sequence, though the MAP approximation in the previous set of simulations can introduce order effects.

We can similarly quantify the degree of predictability of X by evaluating the entropy of the sequence, defined $H(X) = -\sum_{\mathcal{P}} p_x \log_2 p_x$. Here, we define a sequence $X^{(n)}$ to allow us to monotonically decrease $H(X)$ with n. Let $X^{(n)}$ be the sequence $A^{(n)}BCD$, where n denotes the number of times A appears in the sequence. For example, $X^{(1)}$ is the sequence $ABCD$ and $X^{(3)}$ is the sequence $AAABCD$. For simplicity, we assume the probability distribution $\mathcal{P}$ over the ensemble $\mathcal{K}$ is exchangeable and that the probabilities over the members of its ensemble are proportional to their frequency in the sequence such that $p_k = N_k/||X||$ where N_k is the number of times k appears in sequence X. Consequently, the entropy of the sequence $X^{(1)}$ is $H(X^{(1)}) = 2$ bits and the entropy of the sequence $X^{(3}$ is $H(X^{(3)}) \approx 1.79$bits. As n approaches infinity, the entropy $H(X^{(n)})$ asymptotically approaches 0 bits. Intuitively, as A is repeated more often in the sequence, the sequence is more predictable (lower entropy). It is important to note that the sequence predictability does not depend on order. Because the CRP is exchangeable, it will have the same predictive error for the sequences ABCDABCD and ADBCDBAC. Order-dependent predictability is beyond of the scope of the current work.

We evaluated the CRP on $X^{(n)}$ for $n = [1, 100]$ and compared it to a naïve guess (uniform distribution over $\mathcal{M}$). Because the CRP is parameterized by its tendency to generate a new cluster, the value of its α parameter alters the predictive distribution. To establish an upper limit on the performance of the CRP, we used numerical optimization to determine α for each value of n. In addition, we also evaluated the performance of an agent with a fixed $\alpha = 1$, which we believe is a more accurate reflection of a generalizer in an unknown environment. As expected, as we increase the structure of the sequences (lower entropy), the CRP advantage over a naïve guess increases (S2 Fig, left, green line). Similarly, the optimal value of α declines with the sequence structure, such

that it is more advantageous to cluster as the sequence becomes more predictable (S2 Fig, right). However, this benefit is minimal for very unstructured sequences, and for fixed values of α, clustering for highly unstructured sequences ($H(X) \lesssim 1.45$bits) yields worse CRP performance compared to a naïve guess.

S2 Fig. Performance of the CRP as a function of task domain structure. *Left*: Relative information gain of a naïve guess over the CRP as function of sequence entropy for a CRP with an optimized alpha parameter (green) or fixed at $\alpha = 1.0$. *Right*: Optimized alpha value (log scale) as a function of sequence entropy.

References

1. Aldous DJ. Exchangeability and related topics. In École d'Été de Probabilités de Saint-Flour XIII—1983 1985 (pp. 1-198). Springer, Berlin, Heidelberg.
